# Burden of Misconception in Sexual Health Care Setting: A Cross-Sectional Investigation among the Patients Attending a Psychiatric Sex Clinic of Bangladesh

**DOI:** 10.1155/2017/9827083

**Published:** 2017-06-27

**Authors:** S. M. Yasir Arafat, Srijony Ahmed

**Affiliations:** Department of Psychiatry, Bangabandhu Sheikh Mujib Medical University, Dhaka, Bangladesh

## Abstract

**Background:**

Bangladesh is a country in South Asia with about 160 million people and achieved health related Millennium Development Goals (MDG) significantly. But sexual health is still an untapped issue with predominant myths and misconception.

**Objective:**

We aimed to look into the proportions of patients attending sexual health care services due to misconceptions.

**Methods:**

The descriptive, cross-sectional study was conducted among 110 patients attending Psychiatric Sex Clinic (PSC) of Bangabandhu Sheikh Mujib Medical University. Respondents were included in the study with convenient sampling from November 2016 to March 2017. Data were collected through face-to-face interview with semistructured preformed, pretested questionnaire and analyzed by SPSS software 16.0 version.

**Results:**

Most of the patients (93%) were male, 60% were married, 62% were urban habitant, 42% were under grade 10, and 33% were service holder. Total 55% of the patients had misconceptions and 29% visited only for misconception; 14% had Premature Ejaculation; and 12% had desire disorder. 32% of the patients had psychiatric disorders and among them depression was most common, 13%.

**Conclusion:**

Positive openness in sexual health and appropriate strategy should be taken to improve the quality of sexual life as well as reduce the misconception in the people of Bangladesh.

## 1. Introduction

World Health Organization (WHO) defines sexual health as “a state of physical, emotional, mental, and social well-being in relationship to sexuality; it is not merely the absence of disease, dysfunction, or infirmity [1–3].” Positive sexual health requires respectful approach to sexuality with possibility of having pleasurable and safe sexual experiences with those who are free of coercion, discrimination, and violence [1, 2]. Sexuality, partner role, distribution of dysfunctions, perception, and expression of sexual complaints often vary in the aspects of different cultures, societies, countries, religions, and other such aspects. Dysfunctions may prevail globally but expression, presentation, help seeking behavior, and approaches to correct the dysfunctions often vary. Bangladesh is a developing country in South Asia with about 160 million people and achieved health related Millennium Development Goals (MDG) significantly [4]. The literacy rate is increasing day by day and currently 61.0% of the population have 15 years and above age group education year [4]. But the health literacy state is still in dearth in Bangladesh and misconceptions direct the population to pursue abnormal health seeking behaviors [2, 5]. Sexuality is still a covert issue in Bangladesh and people usually hesitate to start talk regarding sex [3, 5]. Traditional healers and vaids contribute to disseminating different sexual misconception in addition to the existing cultural and religious myths [5, 6]. There is dearth of research as well as publications to assess the local misconception and to remove the myths. In addition there is no specialized services center to deal with sexual dysfunctions [3]. So, sufferers are in grave situation as they find difficulties to reach the appropriate services provider and to get relief from the problem. Authors aimed to look into the proportions of patients attended for sexual health care services due to misconceptions in a tertiary care university hospital of Bangladesh. It was also aimed to look into the distribution of misconceptions in relation to the demographic variables.

## 2. Methods

### 2.1. Place of the Study

Bangabandhu Sheikh Mujib Medical University (BSMMU) is the only medical university for about 160 million population of Bangladesh to date. Also, Psychiatric Sex Clinic (PSC) is the single specialized psychiatric service based sex clinic in the country. The clinic is run under the Department of Psychiatry, BSMMU, headed by an associate professor of psychiatry. Only referred patients are serviced here and there is allocated time to attend the clinic in a single day of the week. The usual turnover is about 15–20 patients per session of the clinic. In Bangladesh the patients with sexual complaints usually visit Dermatology and Venereology Department and the referral system is not well defined yet. Most of the referrals are from the Psychiatry Outpatient Department (OPD), and due to regular activity and awareness programs there are few referrals from other disciplines such as Dermatology & Venereology Department.

### 2.2. Methods

The descriptive, cross-sectional observational study was conducted among 110 patients attending PSC of BSMMU. Respondents were chosen with convenient purposive sampling within the period of November 2016 to March 2017. Data were collected through face-to-face interview with semistructured preformed, pretested questionnaire by the authors themselves and analyzed by SPSS 16.0 software.

### 2.3. Questionnaire

A semistructured questionnaire was formed to conduct the study. In demographic part age, sex, residence, nuptial status, educational status, occupation, source of referral, and history of substance abuse were included. And chief complaints, duration of illness, psychiatric comorbidity, cooccurring medical history, and other notable findings of clinical interview were included in the diseases part.

## 3. Results

The mean age of the respondents was 30.42 ± 8.13 years (mean ± SD) and range was 17–55 years. Majority of the patients (52%) belonged to 26–35-year group. 93% of the respondents were male, 60% were married, 63% were urban habitant, 42% studied less than grade 10 (S.S.C.), and 34% belonged to service holder as an occupational class (Table 1).

Among the respondents 60 (54.44%) patients had misconceptions (MC) regarding sexual health, function, illness, and disorders as well as treatments. 29% of the patients visited for the problems only related to misconceptions and others visited for misconception along with sexual dysfunctions comorbidly (Table 2). Among the sexual dysfunctions Premature Ejaculation was most common (13.64%) in males and among the female sexual dysfunctions, Female Sexual Arousal/Interest Disorder (FSAID) was the most common (7.27%) (Table 2). All the female respondents were found to have FSAID. This finding can be explained by the cultural pattern of the females in Bangladesh where females consider sex as a minor consideration. Few patients presented with global dysfunctions along with misconception (Table 2).

**Figure 1:**
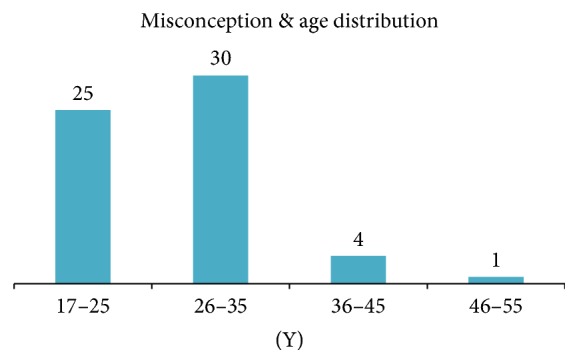
Distribution of misconception with age among respondents (*n* = 60).

**Figure 2:**
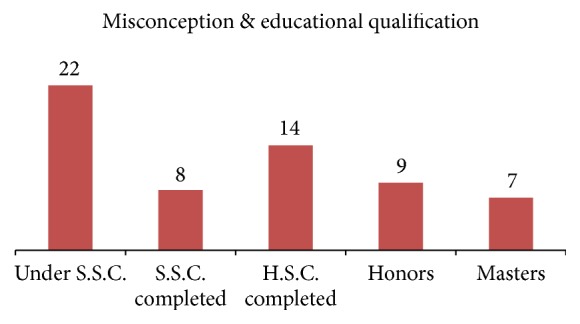
Distribution of misconception with educational qualification among the respondents (*n* = 60).

Among the psychiatric disorders the most common diagnosis was Major Depressive Disorder (MDD) (13.64%) and among the general medical condition infertility was found as an important diagnosis (5.45%) (Table 3). Among the respondents 9 (8.18%) patients presented with the history of substances abuse (Table 3). The result revealed that misconception prevailed in the younger age as 91% of the patients with misconceptions were under 35 years and majority were in the 26–35-year group (50%) (Figure 1). Exploration of misconception with educational qualification revealed that misconception was also found in the well educated persons and even in masters holders (11.67%) but it was found the most in under S.S.C. (under grade 10) education group (36.67%) (Figure 2). But the ratio of misconception in educational qualification to the total sample educational qualification is nearly the same (Table 1 and Figure 2).

## 4. Discussion

It was aimed at seeing the burden of misconception in terms of proportion of patients attending a Psychiatric Sex Clinic in Bangladesh because based on clinical observation authors thought misconceptions might be a major cause of sexual service consumption. The result revealed that majority (82.73%) of respondents were below the 35-year age group; more than half of the study population had study less than S.S.C. (Under grade 10), and 36.36% were unmarried (Table 1). Young unmarried less educated people are visiting more and misconception was found to be the major cause for visiting and these results are aligned with similar article in neighboring country as well as few reported articles in the same country regarding misconceptions [5–8]. The results revealed that total of about 55% patients visited for their problems along with sexual misconception and about 29% patients visited for their problem caused only for their misconception (Tables 2 and 3). Previous available studies and articles revealed similar pattern of presentation and services consumptions [6–8]. Arafat SMY mentioned Dhat syndrome is a frequently presented but underaddressed misconception in the primary care level of Bangladesh and the syndromal presentation is well recognized in this culture [6–8]. Depression was found as most common psychiatric comorbidity and the result can be explained by the pathophysiology and symptom presentation of depression [9, 10]. The distribution of misconception was found more in the younger age (Figure 1) and that is supported by previous literature of Dhat syndromes and/or cultural bound syndrome [6–8]. Dhat syndrome is a culture bound syndrome of the Indian subcontinent which is characterized by preoccupation with loss of “Dhat” (semen) and attribution of different physical and psychological symptoms [6–8]. Dhat syndrome is placed in the classificatory system (DSM-IV and ICD-10) earlier, but in DSM 5 it is placed in the glossary section [6, 11–13]. Interestingly attitude to the misconceptions was not found to be changed with educational achievements among the study population. It was prevalent nearly in the same ratio to the total sample with the educational status in all educational groups (Table 1 and Figure 2). This situation could be explained by considering that the study population were the referred clinical samples, not the general population. However, that might be due to lack of systemized and coordinated education regarding sex in the academic curricula. The high prevalence of misconceptions could be explained by cultural covertness regarding sex [3] and lack of poor health literacy in curricula [4] and samples were taken from clinical populations, single centered study, preexisting myths [5, 6], religious misperceptions [3, 5], and other such factors. Creating awareness regarding sexual health as a part of the holistic health, incorporating sexual health education in school curriculum, preventing the unauthentic dissemination of sexual myths, specialized services center, and proper referral can be considered as further approach in regard to minimizing the services consumptions due to misconception.

## 5. Conclusion

Though larger sample size and multicenter involvement would be better to generalize the results, the study revealed that misconception consumes a major part of resources in a country like Bangladesh. Positive openness in sexual health and appropriate strategies are time demanded to raise the awareness against the myths and misconception as well as raise the overall quality of life of the people of the country.

## Figures and Tables

**Table 1:** Distribution of demographic variables of the respondents (*n* = 110).

Demographic variable	Frequency	Percentage
*Age in years*		
17–25	34	30.91
26–35	57	51.82
36–45	11	10.00
46–55	8	7.27

*Sex*		
Male	102	92.73
Female	8	7.27

*Marital status*		
Single	40	36.36
Married	66	60.00
Separated	1	0.91
Divorced	3	2.73

*Habitat*		
Urban	69	62.73
Rural	41	37.27

*Education*		
Under S.S.C.	46	41.82
S.S.C. completed	12	10.91
H.S.C. completed	23	20.91
Honors	16	14.55
Masters	13	11.82

*Occupation*		
Farmer	4	5.41
Service holder	25	33.78
Professionals	6	8.11
Unemployed	5	6.76
Businessman	10	13.51
Student	12	16.22
Housewife	6	8.11
Abroad	1	1.35
Day laborer	5	6.76

**Table 2:** Distribution of problems among the respondents (*n* = 110).

Problems of the patients	Frequency	Percent
Misconception (MC)	32	29.09
Premature Ejaculation (PE)	15	13.64
MC + PE	13	11.82
Erectile Dysfunction (ED)	9	8.18
MC + ED	9	8.18
Female Sexual Arousal/Interest Disorder (FSAID)	8	7.27
PE + ED	6	5.45
Hypoactive Sexual Desire Disorder (HSDD)	5	4.55
Homosexuality	3	2.73
MC + PE + HSDD + ED	2	1.82
MC + PE + ED	2	1.82
Orgasmic disorder	2	1.82
MC + homosexuality	2	1.82
HSDD + ED	2	1.82
*Total*	*110*	*100*

**Table 3:** Distribution of comorbid disorders among the respondents (*n* = 110).

Psychiatric disorders	Frequency	Percent
MDD	15	13.64
Schizophrenia	4	3.64
GAD	3	2.73
Personality disorder	2	1.82
OCD	2	1.82
Social phobia	2	1.82
Bipolar 1	2	1.82
Gender identity disorder	1	0.91
Delusional disorder	1	0.91
Panic disorder	1	0.91
Somatic symptom Disorder	1	0.91
*Total*	*34*	*30.91*

Other disorders	Frequency	Percent

Infertility	6	5.45
HTN	3	2.73
IBS	3	2.73
Hypothyroidism	2	1.82
DM	1	0.91
Epilepsy	1	0.91
Thyrotoxicosis	1	0.91
*Total*	*17*	*15.45*

*Substance*	*Frequency*	*Percent*

Yes	9	8.18
No	101	91.82
*Total*	*110*	*100*

MDD: Major Depressive Disorder; GAD: Generalized Anxiety Disorder; OCD: Obsessive Compulsive Disorder; HTN: hypertension; IBS: irritable bowel syndrome; DM: diabetes mellitus.

## Abbreviations

WHO: World Health Organization
MDG: Millennium Development Goals
PSC: Psychiatric Sex Clinic
BSMMU: Bangabandhu Sheikh Mujib Medical University
OPD: Psychiatry Outpatient Department
MDD: Major Depressive Disorder
GAD: Generalized Anxiety Disorder
OCD: Obsessive Compulsive Disorder
ICD: International Statistical Classification of Diseases
DSM: Diagnostic and Statistical Manual of Mental Disorders.

## References

[1] Thomas H. N., Thurston R. C. (2016). A biopsychosocial approach to women's sexual function and dysfunction at midlife: a narrative review. *Maturitas*.

[2] Edwards W. M., Coleman E. (2004). Defining sexual health: a descriptive overview. *Archives of Sexual Behavior*.

[3] Ahsan M. S., Arafat S. M. Y., Ali R., Rahman S. M. A., Ahmed S., Rahman M. M. (2016). Sexual history taking competency: a survey among the clinicians in Bangladesh. *International Journal of Psychiatry*.

[4] Saleh Uddin M., Mashrur Ahmed S. R. (2016). Does mind exist in physician's mind? a brief phone survey in Bangladesh. *International Journal of Neurorehabilitation*.

[5] Arafat S. M. Y. (2017). Abnormal health believes with frequent presentations: ethnographic observation from primary care of Bangladesh. *International Journal of Perceptions in Public Health*.

[6] Arafat S. M. Y. (2017). Dhat syndrome: culture bound, separate entity, or removed. *Journal of Behavioral Health*.

[7] Deb K. S., Balhara Y. P. S. (2013). Dhat syndrome: a review of the world literature. *Indian Journal of Psychological Medicine*.

[8] Grover S., Gupta S., Avasthi A. (2015). Psychological correlates and psychiatric morbidity in patients with Dhat syndrome. *Indian Journal of Psychiatry*.

[9] Cowen P., Harrison P., Burns T. (2012). *Shorter Oxford Textbook of Psychiatry*.

[10] Kermode M., Herrman H., Arole R., White J., Premkumar R., Patel V. (2007). Empowerment of women and mental health promotion: a qualitative study in rural Maharashtra, India. *BMC Public Health*.

[11] American Psychiatric Association (2000). *Diagnostic and Statistical Manual of Mental Disorders*.

[12] World Health Organization (2004). *International Statistical Classification of Diseases and Health Related Problems ICD-10*.

[13] American Psychiatric Association (2013). Glossary of cultural concepts of distress. *Diagnostic and Statistical Manual of Mental Disorders*.

